# Metabolic Risk Profile among Overweight and Obese Lactating Women in Sweden

**DOI:** 10.1371/journal.pone.0063629

**Published:** 2013-05-07

**Authors:** Anna Winkvist, Fredrik Bertz, Lars Ellegård, Ingvar Bosaeus, Hilde K. Brekke

**Affiliations:** Department of Internal Medicine and Clinical Nutrition, The Sahlgrenska Academy, University of Gothenburg, Gothenburg, Sweden; Queensland University of Technology, Australia

## Abstract

**Background:**

Obesity and cardiovascular diseases are increasing globally and any association between reproduction and these conditions is of concern. Unfortunately, little is known about normal levels of metabolic risk factors in women of different body mass index throughout the reproductive cycle. This study is one of the first to describe the metabolic risk profile of lactating overweight or obese women at 8–12 weeks postpartum.

**Methods:**

During 2007–2009, 66 overweight or obese Swedish lactating women without known diseases underwent detailed measurements of their metabolic profiles, dietary intake and general health before entering a lifestyle intervention trial. Baseline measurements took place between 8–12 wk postpartum. Almost all women were exclusively breastfeeding their term infants.

**Results:**

The women were regarded as healthy, as reflected in the absence of diagnosed diseases, their own perceptions and in normal hemoglobin, albumin and fasting plasma glucose values. Four women were diagnosed with metabolic syndrome. In these cases, underlying conditions included large waist circumference, low HDL cholesterol values, high triglyceride values and relatively high blood pressure. The metabolic profile differed between overweight and obese women; obese women had significantly higher levels of fasting insulin (p = 0.017), borderline higher HOMA values (p = 0.057) and significantly higher triglyceride values (p = 0.029), as well as larger waist and hip circumferences (p<0.001 and p<0.001). However, no significant differences between overweight and obese women were detected for LDL or total cholesterol levels. Overweight and obese women reported similar total energy and macronutrient intakes, but obese women tended to be less physically active (p = 0.081).

**Conclusions:**

Among generally healthy lactating women, obesity as compared to overweight is associated with increased metabolic risk. This cut-off is thus important also in the early postpartum period, and obesity among these women should warrant proper health investigation. Macronutrient intake did not differ between the groups and, hence, cannot explain these differences.

**Trial registration:**

ClinicalTrials.gov Identifier: NCT01343238

## Introduction

On a worldwide basis, the prevalence of overweight and obesity has increased alarmingly during the last decades [1]. These higher rates of overweight and obesity are also seen among pregnant women [2]. In the US, 60% of women of reproductive age are overweight or obese, i.e., it has become the most prevalent condition [3]. As rates of obesity increase worldwide, so do the risks for obesity related diseases like cardiovascular diseases (CVD) and diabetes. Epidemiological evidence exists for an increased risk for CVD and the metabolic syndrome with childbearing per se, with or without a weight increase as intermediate link [4]–[7]. From a life course perspective, for women, any association between reproduction and metabolic risk factors is important [8].

Pregnancy imposes great stress on the metabolism. In parallel with dramatic changes in hormone levels and increasing insulin resistance, blood lipids rise by 40% (high-density lipoprotein cholesterol) to 300% (triglycerides) from before pregnancy to the end of gestation in normal weight women [9]. Little is known about the lipid metabolism of overweight and obese women during pregnancy. However aberrations in blood lipids in the third trimester have been observed in those with higher body mass index (BMI) compared to normal weight women [10], [11]. Postpartum metabolism is even less studied. Longitudinal data from normal weight women show that lipid metabolism gradually normalizes postpartum, depending on the degree of lactation [12]. The importance of degree of overweight for the metabolic state postpartum in lactating women is unknown.

The aim of this paper is to compare the metabolic risk profile of generally healthy lactating overweight versus obese women at 8–12 weeks postpartum.

## Methods

### Subjects

During 2007–2009, pregnant women who were overweight or obese (BMI 25–34.9 kg/m^2^) before pregnancy were invited to join a randomized trial postpartum, aimed at weight loss through a 12 wk intervention with either dietary restriction, physical exercise or both. Women were recruited through advertisements at 15 antenatal care clinics in the region of Gothenburg, the second largest city of Sweden with 7700 deliveries per year. In total, 71 women met the inclusion criteria (non-smoker, singleton term delivery, birth weight>2500 g, intention to breastfeed for 6 months, and no suspected or diagnosed illness on the part of the mother or infant) and agreed to participate. Among these, 66 women completed the baseline measurements.

### Ethics statement

The study received full ethical approval by the regional ethics board in Gothenburg, Sweden. All participants were informed about study protocol and signed an informed consent sheet.

### Study design

This study reports on results of the baseline measurements among the 66 women, who thereafter entered a randomized controlled trial (LEVA, Livsstil vid Effektiv Viktminskning under Amning; Lifestyle for effective weight loss during lactation).

### Measurements

Baseline measurements were initiated between 8–12 wks postpartum and were carried out at the laboratory of the Dept Internal Medicine and Clinical Nutrition, University of Gothenburg, Sweden, after an overnight fast. Anthropometric measurements included body weight to the nearest 0.1 kg in light underclothing on an electronic scale (MC 180 MA, Tanita, Tokyo, Japan), height using a wall-mounted measuring stick and waist-hip ratio. Waist and hip circumferences were both assessed with a flexible, non-stretch tape measure with a spring loaded handle; waist circumference mid-way between the tenth rib and the iliac crest and hip circumference at the widest point over the buttocks.

Daily step count was measured during 5 consecutive days with a SenseWear® Pro2 Armband, version 6.03 and InnerView® Professional software, version 5.1 (BodyMedia, Inc, Pittsburgh, PA). Armband placement was on the women’s right upper arms. Cardiovascular fitness was measured with a bicycle ergometer (EBIKE Comfort, GE Medical System, Milwaukee, USA) in upright position until exhaustion and reaching a minimum respiratory exchange ratio of 1. Breath-by-breath gas analysis by Ergospirometry was applied (Jaeger Oxycon Pro, Viasys Healthcare GmbH, Hoechenberg, Germany). Initial workload was set at 40 W, with increments of 15 W/minute. During the test, heart rate, a 12-lead electrocardiogram, subjective symptoms and perceived exertion were recorded. The highest oxygen uptake measured during the test was used for maximum oxygen uptake (VO2 max). Before the test, the women rested in the supine position for 5 min and blood pressure was measured using the auscultatory method on the right arm. A fasting venous blood sample was taken by trained staff and sent to the accredited Laboratory of Clinical Chemistry, Sahlgrenska University Hospital, Gothenburg, Sweden, for analyses of total cholesterol, low-density lipoprotein cholesterol (LDL), high-density lipoprotein cholesterol (HDL), triglycerides, high sensitive C-reactive protein (CRP), hemoglobin (Hb), albumin, glucose, insulin, insulin-like growth factor 1 (IGF-1), thyroxine (free T4), thyroid stimulating hormone (TSH). Two markers of inflammation (IL-6 and TNF-α) were measured in serum with the Human Proinflammatory 9-plex Ultrasensitive Kit in a SECTOR 2400 Imager (Meso Scale Discovery, Gaithersburg, MD) at The Wallenberg Laboratory for Cardiovascular and Metabolic Research, University of Gothenburg, Sweden. Homeostasis model assessment (HOMA) was calculated as (glucose * insulin/22.5) [13]. The metabolic syndrome was defined according to the criteria suggested by NCEP ATP III as having at least three of the following characteristics: fasting blood glucose≥6.1 mmol/L, blood pressure≥130/85 mmHg, triglycerides≥1.7 mmol/L, HDL cholesterol<1.30 mmol/L or waist circumference>88 cm (NCEP 2005). No women used blood pressure or cholesterol lowering drugs.

Women were provided with an electronic kitchen scale and instructed to weigh and record all foods and beverages (to the nearest 1 g) consumed for 4 consecutive days. Dietary intake was calculated with Dietist XP software (version 3.2, Kost och Näringsdata, Bromma, Sweden), using the Swedish Food Database 2010, and data from food manufacturers.

### Statistical analyses

Mean (SD) values for all 66 women are shown for normally distributed continuous variables, median and interquartile range for non-normally distributed continuous variables and proportions for categorical variables. Based on measured weight and height at baseline, women were categorized into overweight (n =  32) or obese (n = 34). These two groups were compared using independent sample t-test, Mann-Whitney U-test and Chi-square test. For inflammation markers, values>+3 SD were removed before analyses (one individual). Significance level was set to 0.05. All analyses were performed in SPSS version 19.0 (IBM, Somers, NY, USA).

## Results

The women in the study represented a relatively well-educated sample; most women (73%) had more than 3 yrs of education beyond upper secondary school only 20% had 3 yrs or fewer beyond upper secondary school and only 7% had completed upper secondary school only. The majority (96%) of the women were married or cohabitating and only 4% were single. No women had smoked during the recent pregnancy, although 25 had stopped smoking shortly before (min 2 mo; max 2 yrs). For 52% of the women, the recent newborn was their first child, whereas for 42% of the women it was the second child and for 6% it was the third child. In agreement with the inclusion criteria, all infants (50% female) had been born at term, with a mean birth weight of 3.8+0.5 kg and a mean birth length of 51.1+2.2 cm. Almost all women (94%) were exclusively breastfeeding their infants at the measurement session at 8–12 wks postpartum. The four women (three overweight and one obese) who also offered their infants complementary feeds in the form of infant formula, provided these in the amount of 74.0+47.0 kcal/d (min 30.0 and max 140.0 kcal/d).

Self-reported pre-pregnancy weight was 82.8+8.9 kg (min 67.0, max 109.0), corresponding to a mean pre-pregnancy BMI of 29.1 (±2.8), with 68% being categorized as overweight and 32% as obese before pregnancy. In total, 47% of the women were categorized as overweight both pre-pregnancy (self-reported weight) and 8–12 wk postpartum (measured weight), 21% were categorized as overweight pre-pregnancy and as obese 8–12 wk postpartum, 2% were categorized as obese pre-pregnancy and as overweight 8–12 wk postpartum, and 30% were categorized as obese at both time points.

In Table 1, anthropometric and lifestyle characteristics at 8–12 wk postpartum are shown for the group as a whole as well as for women classified as overweight or obese at 8–12 wk postpartum separately. In addition to the expected significant differences in weight and BMI between the two groups, overweight women had significantly higher VO2 max than obese women when expressed as ml/kg/min (*p* = 0.001) but not when expressed as l/min (*p* = 0.648). Further, overweight women tended to demonstrate higher physical activity than obese women, measured as daily steps (*p* = 0.081). Overall, 54.2% of the women had≥8000 steps/d; among overweight women this proportion was 65.5% and among obese 43.3% (*p* = 0.087). Total daily energy intake and macronutrient composition of the diet were basically identical between the two groups.

**Table 1:** Anthropometric and lifestyle characteristics of overweight and obese Swedish women at 8–12 wk postpartum.

	All	Overweight	Obese	Overweight vs obese^1^
	N = 66	N = 32	N = 34	
Age (yr)	33.0+4.2^2^	33.9+4.0	32.2+4.2	0.097
Height (cm)	168.6+6.6	169.1+6.5	168.2+6.6	0.592
Weight (kg)	86.2+9.7	79.8+7.5	92.3+7.5	<0.001
Body mass index (kg/m^3^)	30.3+2.9	27.9+1.3	32.6+1.8	<0.001
Energy intake (kcal/day)	2634+486	2655+498	2614+480	0.736
Protein (% of total energy)	15.4+2.3	15.4+2.3	15.4+2.3	0.948
Carbohydrates (% of total energy)	48.5+5.7	48.5+5.3	48.5+6.1	0.948
Fat (% of total energy)	35.8+5.6	35.9+5.5	35.7+5.7	0.870
Alcohol (% of total energy)	0.3+0.6	0.2+0.4	0.4+0.8	0.301
Physical activity (steps/day)^3^	8948+3063	9656+3388	8264+2588	0.081
VO2 max (ml/kg/min) 4	25.4+3.8	27.0+3.7	23.9+3.4	0.001
VO2 max (l/min)^4^	2.2+0.3	2.2+0.3	2.2+0.3	0.648

1 *p*-value, independent sample t-test.

2 Mean+SD.

3 N =  29+30.

4 VO2 max, maximum oxygen uptake, N = 31+34.

In Table 2, the characteristics included in the definition of the metabolic syndrome are exhibited, both as mean values and as proportions outside the cut-off values applied. In addition, mean values of related metabolic risk factors are demonstrated. In total, 4 (6.2%) of the women were classified as having metabolic syndrome; 1 (3.1%) overweight women and 3 (8.8%) obese women (*p* = 0.348). The overweight woman with metabolic syndrome fulfilled three of the underlying diagnostic criteria: high blood pressure, low HDL values and large waist circumference. Among the three obese women with metabolic syndrome, one fulfilled the same three criteria, one in addition had high triglyceride values, and one had high triglyceride values, low HDL values and large waist circumference.

**Table 2:** Metabolic syndrome and its components and related metabolic risk factors among overweight and obese Swedish women at 8–12 wk postpartum.

	All	Overweight	Obese	Overweight vs obese
	N = 66	N = 32	N = 34	
Proportion with metabolic syndrome (%)^1^	6.2	3.1	8.8	0.348^2^
Fasting plasma glucose (mmol/L)	4.55+0.42^3^	4.49+0.44	4.61+0.40	0.260^2^
≥6.1 mmol/L (%)	0	0	0	
Fasting serum insulin (mU/L)	6.16+3.35	5.15+2.26	7.11+3.93	0.017^2^
HOMA (md, 1^st^–3^rd^ quartile)	1.06 (0.70–1.56)	0.95 (0.66 –1.23)	1.19 (0.81– 2.22)	0.057^4^
Systolic blood pressure (mmHg) 5	116.1 +11.2	114.8 +10.3	117.3 +12.1	0.371^2^
≥130 mmHg (%)	13.6	9.4	17.6	0.328^6^
Diastolic blood pressure (mmHg) 7	76.0+7.7	75.8+6.9	76.3+8.6	0.822^2^
≥85 mmHg (%)	16.7	9.4	23.5	0.123^6^
Systolic blood pressure≥130 or diastolic				
blood pressure≥85 mmHg (%)^7^	24.2	18.8	29.4	0.312^6^
Triglycerides (mmol/L)^7^	0.79+0.31	0.70+0.22	0.87+0.36	0.029^2^
≥1.7 mmol/L (%)	3.1	0	5.9	0.170^6^
HDL cholesterol (mmol/L)^8^	1.56+0.27	1.56+0.24	1.56+0.29	0.995^2^
<1.3 mmol/L (%)	9.2	6.5	11.8	0.460^6^
LDL cholesterol (mmol/L)^8^	3.07+0.79	3.04+0.88	3.10+0.71	0.757^2^
Total cholesterol (mmol/L)^8^	5.07+0.86	4.97+0.95	5.08+0.76	0.585^2^
Waist (cm)	96.6+8.5	91.5+6.0	101.4+7.8	0.000^2^
≥88 cm (%)	87.9	78.1	97.1	0.019^6^
Hip (cm)	113.7+7.3	109.0+5.3	118.2+6.1	<0.001^2^
Waist-hip ratio	0.85+0.06	0.84+0.05	0.86+0.08	0.208^2^

1 Defined according to US National Cholesterol Education Program ATP III (NCEP; Circulation 2005) as at least three out of the following: fasting plasma glucose≥6.1 mmol/L, blood pressure≥130/85 mmHg, triglycerides≥1.7 mmol/L, HDL cholesterol<1.30 mmol/L, or waist circumference≥88 cm. No women used blood pressure or cholesterol lowering drugs.

2 p-value: independent sample t-test.

3 Mean+SD unless indicated.

4 p-value: Mann-Whitney U test.

5 N = 32+33.

6 *p*-value: Chi-square test.

7 N = 32+31.

8 N = 31+34.

None of the women in the full sample had high fasting plasma glucose values, and none of the women had high blood pressure when defined as systolic blood pressure≥140 mm Hg or diastolic blood pressure≥90 mmHg (data not shown), likely reflecting our inclusion criteria. Most women had high waist circumference, perhaps reflecting the postpartum state.

When overweight and obese women were compared, obese women had significantly higher fasting insulin values than overweight women (*p* = 0.017), borderline higher HOMA values (*p* = 0.057), significantly higher triglyceride values (*p* = 0.029) and significantly larger waist and hip circumferences (*p*<0.001 and *p*<0.001) (Table 2).

Few women overall had low Hemoglobin, albumin or thyroxine values, nor high TSH values and there were no differences between overweight and obese women on these accounts (Table 3). However, obese women had significantly higher CRP values than overweight women (*p* =  0.002; Table 3), and also a higher proportion of women with elevated CRP levels (*p* = 0.026). Still, there were no significant differences between overweight and obese women in levels of the two additional markers of inflammation analyzed (data not shown). Serum levels of IGF-1 were normal in all women.

**Table 3:** Clinical indicators of health among overweight and obese Swedish women at 8–12 wk postpartum.

	All	Overweight	Obese	Overweight vs obese
	N = 66	N = 32	N = 34	
Hemoglobin (g/L)	130.0+8.0^1^	129.5+7.9	130.5+8.2	0.616^2^
<117.0 g/L (%)	3.0	3.1	2.9	0.965^3^
Albumin (g/L) 4	38.8+2.1	38.7+2.2	38.8+2.0	0.921^2^
<36.0g/L (%)	3.1	3.2	2.9	0.947^3^
Thyroxine (pmol/L)^4^	14.7+2.4	14.7+2.7	14.7+2.2	0.991^2^
≥22pmol/L (%)	1.5	3.2	0	0.291^3^
Thyroid stimulating hormone (mlU/L)^4^	1.6+0.8	1.6+0.8	1.6+0.9	0.871^2^
≥4.2 mlU/L (%)	0	0	0	
C-reactive protein (mg/L)^4,5^	3.07+2.62	2.06+1.73	4.01+2.98	0.002^2^
≥5.0 mg/L (%)	21.5	9.7	32.4	0.026^3^
Insulin-like growth factor 1 ( µg/L)	149.0+35.7	150.4+37.1	147.7+34.8	0.759^2^

1 Mean+SD unless indicated.

2 *p*-value: independent sample t-test.

3 *p*-value: Chi-square test.

4 N = 31+34.

5 A woman with CRP of 22.0 and signs of breast engorgement and hence local inflammation was excluded from analysis of CRP and additional inflammation markers.

## Discussion

This is the first study reporting on the metabolic risk profile of overweight and obese women in early lactation, although early postpartum fasting plasma glucose and insulin levels have been reported among obese women with recent gestational diabetes mellitus [14]. The proportion of women of childbearing age who are overweight or obese is increasing at alarming rates worldwide [15]. In the US today, 60% of women 20–39 yrs are overweight or obese [3]. The women in our study were regarded as healthy, as reflected by the absence of diagnosed diseases, by the women’s own perceptions of their health and in the normal values for Hemoglobin, albumin, and fasting plasma glucose. Almost all women were exclusively breastfeeding their infants and on average exhibited a level of physical activity in line with current recommendations (≥8000 steps/d) [16]. Thus, the metabolic profiles of these women reflect the consequences of pregnancy and lactation, together with the consequences of different degrees of fatness in the absence of known diseases.

### Prevalence of the metabolic syndrome

Few metabolic disorders were diagnosed. The relatively young age of our study women likely helped them to metabolically cope with their obesity. Still, four women were defined as having metabolic syndrome—eight percent among those who were obese. This is a low proportion considering that 7 – 57% of women worldwide have metabolic syndrome using the ATPIII definition [17]. Underlying conditions among the women in our study included large waist circumference (88%), high blood pressure (24%), low HDL cholesterol values (9%), and high triglyceride values (3%). Hence, large waist circumference and high blood pressure were more pronounced underlying causes than was the case among white American women aged ≥20 years from the NHANES III (1988–1994), where waist circumference (44%) and low HDL cholesterol (39%) represented the largest contributing factors of metabolic syndrome [18]. The limited contribution of serum lipids to metabolic syndrome in our group may in part be explained by the triglyceride lowering and HDL cholesterol increasing effects of lactation [12], [19].

### Glucose metabolism

At study enrolment, women with diagnosed diabetes were excluded and only one of the enrolled women had experienced gestational diabetes mellitus, for which she had been successfully treated with dietary advice. Among included women, none exhibited impaired fasting glucose (fasting plasma glucose ≥ 6.1,WHO 2006 [20]). This is somewhat surprising, as overweight and obesity are strong risk factors for impaired glucose tolerance in pregnancy [21]. However, Kjos and co-workers noted that among women who had been diagnosed with gestational diabetes mellitus the lactation process affected glucose metabolism positively [19]. Perhaps this effect also accounted for the normalized values among the women in our study.

### The effect of degree of fatness on metabolic risks

The women in our study had recently undergone pregnancy and were currently lactating, states known to strongly influence glucose and lipid metabolism [22]. Still, significant differences in the metabolic risk profile were detected between overweight and obese women, indicating that degree of fatness plays an important role for the development of metabolic risk factors, even in this reproductive state. Obese women had significantly higher levels of fasting insulin, borderline higher HOMA index and significantly higher triglyceride values despite a possible insulin sensitivity enhancing and triglyceride lowering effect of lactation. The higher production of insulin exposes obese women to an increased risk of developing diabetes mellitus, hence demonstrating the importance of preventing overweight or obese women from gaining too much weight during pregnancy [2].

The obese women had significantly higher CRP-values than the overweight women, which is not surprising given that obesity is considered a pro-inflammatory state [23]. However, none of the additional inflammation markers differed between the two groups. This could possibly be explained by a better precision in the analysis of CRP than in the analyses of other markers of inflammation.

No significant differences between overweight and obese women were detected for LDL or total cholesterol levels, in line with findings from other studies of metabolic changes in relation to obesity, i.e., higher triglyceride values and lower HDL cholesterol values, but normal LDL cholesterol values [24].

### The effect of lifestyle on metabolic risks

Overweight and obese women reported similar daily energy intakes and macronutrient compositions of their diet. Hence, differences between the two groups in metabolic risk factors may be less associated with diet and more with the higher weight of the obese group. This would indicate that the obese group would benefit from losing weight through reducing total energy intake, rather than from any specific macronutrient change in the current diet. Obese women tended to be less physically active than overweight women (*p* = 0.081). Still, the fitness of the women in absolute measures (l/min) was similar for the two groups. When expressed per body weight (ml/kg/min), the overweight women who were lighter showed higher values, reflecting the higher proportion of fat-free mass. Almost all women in both groups were exclusively breastfeeding; higher intensity of breastfeeding has otherwise been shown to improve glucose metabolism postpartum [14].

This study had a few limitations. First, the women who volunteered for the study were mostly highly educated, with a motivation to change their lifestyle. Also, no women with a pre-pregnancy BMI over 35 or known illnesses (except allergy and stable hypothyroidism) were included in the study. This limits the representativeness of this group for discussions of prevalence of health conditions and lifestyle characteristics. However, we see no reason why our results from comparisons between overweight and obese subjects would not be generalizable. Important strengths of the study include the precise and state of the art measurements of metabolic risk profile, physical fitness, diet and physical activity.

In conclusion, this is the first time that the metabolic risk profile of overweight or obese lactating women has been described in detail, using precise methodology. Thus, although this was a small study, we provide high quality data indicating that the metabolic risk profile differed in relation to degree of fatness. The target group of the study is a growing group in society; hence information on how to prevent further development of metabolic complications is important. The overweight/obesity cut-off is thus important and useful even early in the postpartum period, and obesity among these women should warrant proper health investigation.
